# 2025 Delphi consensus on robotic ventral mesh rectopexy

**DOI:** 10.1007/s00384-025-05003-8

**Published:** 2025-10-16

**Authors:** Carlo Alberto Schena, Simona Ascanelli, Ugo Grossi, Gaetano Gallo, Francesco Marchegiani, Aleix Martínez-Pérez, Gaetano Pavone, Danila Azzolina, Michel Adamina, Paolo Pietro Bianchi, Gabriele Bislenghi, Andrea Braini, Maria Clotilde Carra, Valerio Celentano, Andrea Coratti, Francesca Da Pozzo, Paola De Nardi, Veronica De Simone, André D’Hoore, Eloy Espin-Basany, Alfredo Genovese, Jim Khan, Leonardo Lenisa, Jacopo Martellucci, Ruby Martinello, Michele Masetti, Marco Milone, Gabriele Naldini, Gianluca Pellino, Pierpaolo Sileri, Angelo Stuto, Pasquale Talento, Mario Testini, Alfredo Tonsi, Carlo Ratto, Nicola de’Angelis

**Affiliations:** 1 Department of Surgery, Fondazione Poliambulanza, Brescia, Italy; 2 Department of Translational Medicine and LTTA Centre, University of Ferrara, Ferrara, Italy; 3 Unit of Robotic and Minimally Invasive Digestive Surgery, Ferrara University Hospital, Via Aldo Moro 8, 44124 Ferrara, Italy; 4 Department of Surgery, Oncology and Gastroenterology - DiSCOG, University of Padova, Padua, Italy; 5 Colorectal Surgery Unit, IRCCS San Raffaele Scientific Institute, Vita-Salute University, Milan, Italy; 6 Unit of Colorectal and Digestive Surgery, DIGEST Department, Beaujon University Hospital (AP-HP), University Paris Cité, ClichyParis, France; 7 Unit of Colorectal Surgery, Department of General and Digestive Surgery, Hospital Universitario Doctor Peset, Valencia, Spain; 8 Department of Surgery, University of Valencia, Valencia, Spain; 9 Unit of Anesthesia and Perioperative Medicine, “F. Miulli” Regional General Hospital, 70021 Bari, Italy; 10 Department of Environmental and Preventive Science, University of Ferrara, Ferrara, Italy; 11 Department of Visceral Surgery and Medicine, Inselspital, Bern University Hospital, Bern, Switzerland; 12 Department of Medical and Surgical Specialties, Faculty of Science and Medicine, University of Fribourg, Fribourg, Switzerland; 13 Department of Surgery, Dipartimento Di Scienze Della Salute, Asst Santi Paolo E Carlo, University of Milan, Milan, Italy; 14 Department of Abdominal Surgery, University Hospitals Leuven, Louvain, Belgium; 15 Department of General Surgery, Azienda Sanitaria Friuli Occidentale (ASFO), Pordenone, Italy; 16 Bowel Disease and Ileoanal Pouch Surgery Centre, Chelsea and Westminster Hospital, London, UK; 17 Division of Surgery and Cancer, Imperial College London, London, UK; 18 Division of General and Minimally Invasive Surgery, Misericordia Hospital, Grosseto, Italy; 19 Policlinico Abano Terme, and Villa Maria, Abano TermeRimini, Italy; 20 Department of Gastroenterological Surgery, San Raffaele Scientific Institute, Milan, Italy; 21 Proctology and Pelvic Floor Surgery Unit, Isola Tiberina Hospital - Gemelli Isola, Rome, Italy; 22 Department of General and Abdominal Surgery, Colorectal Unit, Vall d’Hebrón University Hospital, Universitat Autonoma de Barcelona UAB, Barcelona, Spain; 23 Department of Colorectal Surgery, Portsmouth Hospitals University NHS Trust, University of Portsmouth, Portsmouth, UK; 24 Department of Surgery, Surgery Unit, Pelvic Floor Centre, Humanitas San Pio X, Milan, Italy; 25 Emergency Surgery/Pelvic Floor Center, Careggi University Hospital, Florence, Italy; 26 Collegium Medicum, SAN University, Łodz, Poland; 27 Obstetrics and Gynecology Unit, University Hospital Ferrara, Ferrara, Italy; 28 Department of General Surgery, Santa Maria Della Scaletta Hospital, AUSL of Imola, Imola, Italy; 29 Department of Clinical Medicine and Surgery, University of Naples Federico II, Naples, Italy; 30 Division of Colon and Rectal Surgery, Humanitas University, Rozzano, Milan Italy; 31 Unit of Colonproctology, IRCCS Policlinico San Donato, Milan, Italy; 32 Department of Surgery, Pelvic Floor Center, AUSL-IRCCS Reggio Emilia, Reggio Emilia, Italy; 33 Department of Precision and Regenerative Medicine and Lonian Area, Unit of Academic General Surgery “V. Bonomo, ” University of Bari Aldo Moro, Bari, Italy; 34 Colorectal Surgery Department, Royal Sussex County Hospital, University Hospitals Sussex, Brighton, UK; 35 Department of Advanced Medical and Surgical Sciences, Università degli Studi della Campania “Luigi Vanvitelli”, Naples, Italy

**Keywords:** Robotic ventral mesh rectopexy, Robotic surgery, Minimally invasive surgery, Rectal prolapse, Posterior pelvic floor disorders

## Abstract

**Purpose:**

Robotic ventral mesh rectopexy (RVMR) has emerged as an effective technique for addressing rectal prolapse (RP) and associated pelvic floor disorders. However, variability persists regarding preoperative evaluation, patient selection, and procedural techniques. This Delphi consensus aims to provide evidence-based recommendations to standardize practice, enhance patient outcomes, and address key gaps in the literature.

**Methods:**

Thirty-three experts in RVMR participated in a structured Delphi process. The panel addressed 10 key clinical questions, covering preoperative workup, surgical indications, procedural steps, learning curves, training, and RVMR outcomes. The consensus process was reached through iterative surveys, literature reviews, and a rigorous voting methodology, applying the GRADE approach.

**Results:**

A total of 27 consensus statements were formulated, providing standardized recommendations on patient selection, imaging modalities, surgical technique, and expected clinical outcomes. Concerning surgical technique, the panel addressed variations in trocar placement, robotic instrument selection, and docking strategies. Additionally, consensus statements addressed the role of mesh reinforcement, fixation techniques, and the potential for combined procedures in the treatment of multicompartment pelvic organ prolapse. Of the 27 consensus statements, 3 (11.1%) were supported by moderate-quality evidence, whereas 18 (66.7%) were based on low or very-low-quality evidence and 6 (22.2%) on expert opinion.

**Conclusion:**

This consensus provides a structured, consensus-based framework for clinicians and surgeons trying to address the complexities of RVMR and promote standardization and quality improvement in RP management, while acknowledging that the underlying evidence remains largely low-quality.

**Supplementary Information:**

The online version contains supplementary material available at 10.1007/s00384-025-05003-8.

## Background

Robotic ventral mesh rectopexy (RVMR) has rapidly gained traction as a promising minimally invasive surgical approach for rectal prolapse (RP) and pelvic floor disorders [1]. Initial studies have underscored its safety and efficacy, with potential advantages over the traditional laparoscopic approach, including enhanced ergonomics, precision, and improved visualization [2]. Despite these technical benefits, there remains a lack of consensus and high heterogeneity regarding optimal preoperative workup, patient selection, surgical technique, learning curve, and postoperative outcomes. Advancing in this field requires a comprehensive approach encompassing clinical, technological, and interdisciplinary studies. To address this variability, this Delphi consensus evaluated existing scientific evidence to define optimal surgical decision-making and establish expert-driven recommendations for RVMR.

The evidence base for RVMR remains heterogeneous in design, follow-up, and outcome definitions. Consequently, a number of our recommendations are intentionally general where high-certainty data are lacking. The strength of this Delphi lies in its transparent, concise literature summaries that sit alongside each statement, while explicitly acknowledging areas of uncertainty and priorities for future research.

## Methods

Collaborative work among a group of experts was promoted and aimed to draw recommendations in the practice of RVMR. The process began with three coordinators (NdeA, SA, and CAS) developing a list of ten questions, which were submitted to a steering committee for approval. The steering committee, consisting of experienced colorectal surgeons and methodologists (NdeA, SA, CAS, FM, GP, GG, UG, AMP, DA, MCC), crafted a series of questionnaires addressing critical aspects of RVMR. All ten questions received more than 70% agreement among the steering committee members. These questions were formulated using the PICO format, which defined the patient population, type of intervention, comparison, and outcomes under consideration. Then, a panel of experts in colorectal and pelvic floor surgery with substantial experience in RVMR was subsequently invited to participate in a structured Delphi process. This method systematically collects and synthesizes expert opinions through a series of iterative questionnaires, enabling the development of consensus statements even when high-quality evidence is limited or inconsistent [3–5].

Panelists were invited based on documented clinical expertise in RVMR (annual volume and cumulative caseload), academic productivity, and roles in training/education. To ensure domain-specific expertise and alignment with the initiative’s endorsements, participants were drawn from national (Italian) and international experts affiliated with the endorsing societies: the Italian Club of Robotic Surgery (ICORS), the Società Italiana di Chirurgia Colo-Rettale (SICCR), the Società Italiana di Chirurgia (SIC), and the Società Italiana Unitaria di ColoProctologia (SIUCP). All participants declared potential conflicts of interest and completed the prespecified Delphi rounds.

The Delphi process involved multiple rounds of questionnaires until reaching a consensus. During each round, panelists provided feedback and opinions on the proposed statements, with responses anonymized and aggregated. The results were shared with the panel in subsequent rounds, allowing participants to refine their views based on the collective insights of the group [3–5]. Consensus was defined as at least 70% agreement among panelists for a given statement [6]. Statements that failed to achieve consensus were revised and represented in subsequent rounds until an agreement was reached or a lack of consensus was evident.

The recommendations were developed through a two-step process. First, a systematic review and critical appraisal of the available literature were conducted. Second, evidence-based statements were formulated. Thirty-three experienced robotic-assisted rectopexy surgeons were tasked with drafting responses to the ten questions based on the current literature. For each question, the panel provided a concise discussion supported by a review of the evidence and formulated one or more recommendations (Table 1). Two methodologists (DA and MCC) provided guidance throughout the process. The recommendations were developed using the Grading of Recommendations Assessment, Development, and Evaluation (GRADE) methodology to assess the level of evidence for each bibliographic reference supporting the recommendations (https://www.gradeworkinggroup.org/) [7–12]. Subsequently, a review committee comprising 33 robotic-assisted rectopexy experts independently evaluated the panel written responses. Using the Delphi methodology and an online Google Forms platform, all experts rated each recommendation on a Likert scale from 1 (completely disagree) to 5 (completely agree).

**Table 1:** Key questions and the working groups of experts

*Question 1* *(PICO)*	*What is the optimal clinical, imaging and functional work-up to guide surgical planning for RVMR?*
Team Leader	S. Ascanelli
Experts	A. Braini, J. Martellucci, P. Sileri and A. Tonsi
	• Population: Patients with rectal prolapse • Intervention: Anorectal manometry, RX defecography, and RM defecography before robotic rectopexy • Comparison: Not specified (optional) • Outcome: Accuracy (sensitivity, specificity) of the work-up for surgical planning
*Question 2* *(PICO)*	*What are the indications for RVMR?*
Team Leader	U. Grossi
Experts	S. Ascanelli, G. Naldini, A. Genovese and R. Martinello
	• Population: Patients with rectal prolapse are candidates for surgery • Intervention: Robotic rectopexy • Comparison: Other surgical techniques (e.g., laparoscopy, open) • Outcome: Operative and postoperative outcomes
*Question 3* *(PICO)*	*What is the optimal anesthetic strategy for RVMR?*
Team Leader	G. Pavone
Experts	N. de’Angelis, C.A. Schena, G. Pellino and F. Marchegiani
	• Population: Patients undergoing robotic rectopexy • Intervention: Anesthetic approach • Comparison: Not specified (optional) • Outcome: Intraoperative and postoperative outcomes
*Question 4* *(PICO)*	*What training/experience should the surgeon have before performing RVMR? Is the learning curve for RVMR shorter compared to the learning curve for LVMR?*
Team Leader	F. Marchegiani
Experts	V. Celentano, M. Milone, A. Coratti and A. Martinez-Perez
	• Population: Surgeons in training for robotic rectopexy (residence or fellow or graduated general surgeons) • Intervention: Robotic rectopexy • Comparison: Not specified (optional) • Outcome: Reduction in operative time and improvement in surgical outcomes
*Question 5* *(Technical note)*	*For RVMR, what is the recommended setup regarding the positioning of the trocars, the docking of the robot, and the surgical instruments necessary for the procedure?*
Team Leader	C.A. Schena
Experts	N. de’Angelis, J. Khan, M. Testini, A. Genovese and C. Ratto
*Question 6* *(Technical note)*	*For RVMR, should a prosthetic mesh reinforcement always be recommended? What are the optimal prosthetic materials and fixation techniques for RVMR?*
Team Leader	G. Gallo
Experts	A. D’Hoore, G. Bislenghi, P. Talento, V. De Simone and A. Stuto
	• Population: Patients undergoing robotic rectopexy • Intervention: Use of prosthetic mesh with specific fixation techniques • Comparison: Rectopexy without mesh or with other fixation techniques • Outcome: Short-term and long-term outcomes
*Question 7* *(Technical note)*	*What are the surgical steps to be followed for RVMR?*
Team Leader	C.A. Schena
Experts	G. Naldini, C. Ratto, A. Genovese, A. Martinez-Perez and A. Stuto
*Question 8* *(Technical note)*	*What type of surgical procedure could be combined in case of multicompartment prolapses during RVMR?*
Team Leader	S. Ascanelli
Experts	R. Martinello, P. De Nardi, Lenisa, Da Pozzo and G. Pellino
*Question 9* *(PICO)*	*Does the robotic approach for rectopexy provide short and/or long-term advantages compared with laparoscopy?*
Team Leader	F. Marchegiani
Experts	G. Naldini, A. Martinez-Perez, G. Bislenghi, C.A. Schena, and P. Sileri
	• Population: Patients with rectal prolapse scheduled for surgery • Intervention: Robotic rectopexy • Comparison: Laparoscopic rectopexy • Outcome: Postoperative short-term and long-term outcomes
*Question 10*	*What types of studies and research should be encouraged in the field of robotic surgery for rectal prolapse?*
Team Leader	A. Martinez-Perez
Experts	N. de’Angelis, P. Bianchi, A. Coratti, M. Adamina, D. Azzolina, M. Masetti and E. Espin-Basany

*RVMR* robotic ventral mesh rectopexy, *LVMR* laparoscopic ventral mesh rectopexy

The final consensus statements, along with supporting evidence and rationale, were presented at the “1st Masterclass on Robotic Ventral Mesh Rectopexy” held in Ferrara on January 24, 2025. A comprehensive review of the literature, consensus statements on RVMR, and their Quality of Evidence and Strength of Recommendation are detailed below and summarized in Table 2. The only two statements that did not achieve consensus among the experts after the entire Delphi process are reported in Supplemental Material.

**Table 2:** Summary of the key questions and statements

*Question 1*	*What is the optimal clinical, imaging and functional work-up to guide surgical planning for RVMR?*
1.1: In patients with rectal prolapse candidates for RVMR, a comprehensive evaluation, including physical examination, anoperineal examination, and anoscopy is recommended	
1.2: Additional diagnostic investigations, such as colonoscopy, fluoroscopic defecography, dynamic magnetic resonance defecography, anorectal manometry, anal endosonography, and urodynamic studies may be considered in cases with suspected coexisting pathologies, obstructed defecation symptoms, or impaired anal continence. These tests can also help identify associated anterior pelvic floor support defects (e.g. cystocele, vaginal vault prolapse, and enterocele)	
*Question 2*	*What are the indications for RVMR?*
2.1: RVMR can be indicated and safely performed in patients with primary or recurrent external rectal prolapse	
2.2: RVMR can be indicated and safely performed in patients with internal rectal prolapse presenting with symptoms of obstructed defecation syndrome and/or fecal incontinence, whether or not associated with other anatomical abnormalities (e.g., enterocele, rectocele), and refractory to conservative management	
2.3: RVMR can be indicated and safely performed in patients with isolated rectocele or rectocele associated with enterocele after the failure of conservative management	
*Question 3*	*What is the optimal anesthetic strategy for RVMR?*
3.1: Preoperative evaluation for patients undergoing RVMR should be conducted by anesthesiologists following the current guidelines for the preoperative assessment of non-cardiac surgery patients	
3.2: For analgesia during RVMR, opioid-sparing protocols are recommended. These may include intraoperative lidocaine and ketamine infusions, as well as the use of a transversus abdominis plane block. The use of intrathecal morphine should be avoided due to its associated side effects	
3.3: During the perioperative period, balanced crystalloid solutions are recommended, while normal saline should be avoided. The use of colloids should be minimized and limited for cases of severe hemodynamic instability due to volume loss. The goal should be achieving a near-zero fluid balance. In high-risk patients, advanced hemodynamic monitoring and goal-directed fluid therapy may be considered	
*Question 4*	*What training/experience should the surgeon have before performing RVMR? Is the learning curve for RVMR shorter compared to the learning curve for LVMR?*
4.1: Structured training following national regulations could be suggested to perform RVMR. However, no formal definition of RVMR training currently exists, and further studies are required to ensure the safe adoption of this surgical technique for novice surgeons	
4.2: A potentially shorter learning curve is associated with RVMR compared to LVMR, but the precise threshold cannot be determined due to the limited evidence	
4.3: The learning curve for RVMR should be evaluated considering technical and non-technical skills, previous expertise, availability of a structured program, access to the robotic platform, and caseload	
*Question 5*	*For RVMR, what is the recommended setup regarding the positioning of the trocars, the docking of the robot, and the surgical instruments necessary for the procedure?*
5.1: For the da Vinci multi-arm (X/Xi) Surgical System, common trocar positioning requires four robotic ports and one assistant port, often in a straight or semi-arcuate configuration. The patient cart is usually docked from the patient's left side. Alternative robotic setups can be employed according to the surgeon’s preferences, local resource availability, and hospital economic strategy	
5.2: For the da Vinci multi-arm (X/Xi) Surgical System, common robotic surgical instruments employed for RVMR include EndoWrist monopolar cautery instruments, needle drivers, bipolar instruments, and graspers. The choice of robotic surgical instruments typically relies on the surgeon's preferences, local resource availability, and hospital economic strategy	
5.3: There is insufficient data to recommend a specific robotic setup for robotic platforms other than the da Vinci X/Xi Surgical System	
*Question 6*	*For RVMR, should a prosthetic mesh reinforcement always be recommended? What are the optimal prosthetic materials and fixation techniques for RVMR?*
6.1: The use of a prosthetic mesh for abdominal rectopexy may reduce the risk of recurrence and can be considered in patients with full-thickness rectal prolapse or posterior pelvic floor disorders. However, it is crucial to inform and adequately counsel patients about the potential risks, such as de novo constipation or obstructive defecation syndrome	
6.2: Both biological and synthetic meshes can be considered for RVMR. Synthetic meshes generally offer lower recurrence rates but are associated with higher risks of fistulation and erosions	
*Question 7*	*What are the surgical steps to be followed for RVMR?*
7.1: The autonomic nerve-sparing ventral mesh rectopexy described by D’Hoore represents the reference technique also in case of robotic approach, but technical variations (e.g., without posterior colpopexy, levatorpexy) could be considered according to imaging, clinical evaluation, symptoms, disease characteristics, and patient’s centered outcomes	
7.2: The peritoneum should be incised on the right side of the rectum starting at the level of sacral promontory, medially to the right common iliac artery, down to the pouch of Douglas in an inverted J-form and preserving the homolateral hypogastric nerve plexus and ureter. The dissection should be limited to the anterior rectum and remain superficial, minimizing the risk of potential complications of a posterior rectal dissection	
7.3: The presacral fascia should be exposed on its medial-right side. During the dissection of the sacral promontory, caution should be paid to avoid presacral nerve plexus damage	
7.4: The complete ventral (anterior) dissection of the rectovaginal space in females and rectovesical space in men is a crucial surgical step in RVMR. The dissection should be developed down to the perineal body as distally as possible. After securing the mesh in place, the peritoneum is closed using a continuous absorbable suture covering the mesh along its entire length	
*Question 8*	*What type of surgical procedure could be combined in case of multicompartment prolapses during RVMR?*
8.1: For multicompartmental prolapses, RVMR could be combined with other pelvic organ prolapse reconstructive procedures (e.g. sacrocolpopexy) according to the surgeon’s experience, imaging, and patient characteristics	
8.2: The decision to perform a combined robotic approach for multicompartmental pelvic organ prolapse must be taken by a multidisciplinary team	
*Question 9*	*Does the robotic approach for rectopexy provide short and/or long-term advantages compared with laparoscopy?*
9.1: Robotic and LVMR showed comparable clinical outcomes and can be considered alternative techniques. RVMR may offer some benefits in long-term quality-of-life outcomes	
9.2: RVMR incurs higher overall costs compared to LVMR. Cost-effectiveness should be evaluated in a specific context and include long-term data, considering surgical expertise, team preparation, national regulations, pricing policies, and reimbursement policies	
*Question 10*	*What types of studies and research should be encouraged in the field of robotic surgery for rectal prolapse?*
10.1: Key Research Priorities – Outcome Measures: • Clinical outcomes, such as functional outcomes, recovery time, patient-centered outcomes (e.g., pain, comfort, satisfaction), and quality of life • Cost-effectiveness and risks/benefits evaluation • Safety and delayed adverse events, recurrence rates, and secondary interventions • Patients’ preferences evaluated before the intervention, to integrate this information into the decision-making process, based on the principles of evidence-based medicine	
10.2: Key Research Priorities – methodological considerations: • Multi-center prospective cohort studies and study registries to address clinical and patient-reported outcomes according to the institutional case volume and surgeon’s experience • Prospective studies, observational, and RCTs to assess long-term (> 5 years) outcomes and monitor results, stability/recurrence, over time • In the case of observational and non-randomized studies, controlling the confounding via the propensity score approach or multivariable models is recommended • Advanced big data analytical techniques, including data modeling and machine learning approaches, can improve patient profiling outcomes in scenarios where traditional randomization is challenging or impractical • Patient-reported outcomes and functional (defecatory, urinary, and sexual) outcomes should be assessed in future research by using standardized and validated scoring systems	
10.3: Technological and training research: • Technological innovations (e.g., haptic feedback, AI-guided systems) should be investigated in order to assess whether they are easy to apply and whether they bring significant technical advantages and clinical benefits in the use of robotic platforms for rectal prolapse surgery • Different robotic platforms could be compared for rectal prolapse surgery in order to identify whether they can be considered alternatives or if there are some clear indications for application • Studies assessing the surgeon’s learning curve and the efficacy of training programs integrating robotic rectal prolapse surgery should be conducted	

*RVMR* robotic ventral mesh rectopexy, *LVMR* laparoscopic ventral mesh rectopexy

### Disclaimer

These consensus-driven recommendations are intended as a supplementary resource for planning RVMR. They are not a substitute for clinical judgment but serve as a guide and support for clinicians and surgeons. The content of this publication reflects the collective opinions and recommendations of the contributing authors involved in the Delphi process. While every effort has been made to ensure the accuracy and relevance of the information provided, this document is not intended to replace clinical judgment or individualized patient care. Recommendations should be interpreted within the context of evolving scientific evidence, national governance, clinical expertise, and patient-specific factors. Healthcare professionals are encouraged to consult additional resources and consider their clinical discretion, expertise, and local resources when applying these recommendations in practice.

## Key research questions and statements

**Table:** 

Question no. 1: What is the optimal clinical, imaging, and functional work-up to guide surgical planning for robotic ventral mesh rectopexy?	

### Literature review

Although minimally invasive and RVMR are increasingly utilized for RP in Europe, standardized diagnostic approaches are lacking. Before considering RVMR for posterior pelvic floor disorders, a comprehensive preoperative assessment is crucial. This evaluation should include a thorough history and physical examination, along with selective use of imaging studies and functional tests, to ensure accurate diagnosis and guide surgical planning [13]. While external rectal prolapse (ERP) is often diagnosed clinically, symptomatic internal rectal prolapse (IRP), particularly when presenting with obstructed defecation syndrome (ODS), and other complex pelvic floor disorders, necessitate a more detailed evaluation [13–17].

The importance of preoperative evaluation and specific recommendations have been addressed in several published guidelines for RP management [14–16, 18, 19]. Preoperative evaluation should encompass a thorough review of the patient's presenting symptoms, including bowel habits, continence status, and any associated pelvic floor dysfunction. Specifically, clinicians should inquire about constipation, fecal incontinence, urinary incontinence, dysuria, urinary retention, and symptoms suggestive of anterior compartment prolapse, such as vaginal or uterine prolapse. Physical examination should include a thorough inspection of the perineum with the patient in the lithotomy position to evaluate for ERP, mucosal prolapse, hemorrhoids, rectocele, uterine or vaginal vault prolapse, and urethral hypermobility [14–16, 18, 19]. Furthermore, colonoscopy should be performed in patients with symptoms suggestive of colorectal pathology, such as neoplasms or inflammatory bowel disease or previous complicated diverticulitis, to exclude underlying disease before proceeding with RVMR [14–16, 18, 19].

The extent of preoperative testing and imaging for RVMR should be individualized based on the patient's symptoms and specific presentation of RP or multiorgan POP.

Anorectal manometry and endoanal ultrasonography (EAUS) are valuable for assessing sphincter function and identifying occult sphincter defects, particularly in patients with IRP, ERP, fecal incontinence, or a history of vaginal delivery, pelvic surgery, or trauma [20–22].

Pelvic floor imaging complements multidisciplinary clinical assessment by helping to diagnose and characterize RP and associated anatomical defects (e.g., rectocele, cystocele, enterocele, peritoneocele, sigmoidocele, and descending perineum). While fluoroscopic defecography (FD) has traditionally been considered the gold standard, dynamic magnetic resonance (MR) defecography offers a comprehensive view of the pelvic floor without radiation exposure [23]. The Oxford Rectal Prolapse Grading System should be used to standardize reporting of radiologically confirmed RP [24]. Nevertheless, a 2021 systematic review by van Gruting et al. found that FD had lower specificity compared to other imaging techniques, leading to a higher rate of false-positive diagnoses and potential overtreatment [13]. In cases of concomitant anterior or middle compartment prolapse, dynamic MR defecography is recommended to assess for cystocele, enterocele, and other abnormalities at those levels. Limited access to MR defecography due to cost and equipment availability, along with contraindications for patients with certain medical implants, can restrict its use. Furthermore, MR defecography may not always provide a definitive diagnosis, particularly if a patient cannot evacuate effectively during the examination, potentially obscuring subtle RP or other abnormalities. In such cases, FD, with its dynamic assessment in a more physiological position, may be preferable [13]. Pelvic floor ultrasound, encompassing transperineal (TPUS), endovaginal (EVUS), and endorectal approaches, offers an accessible, cost-effective, and radiation-free method for assessing pelvic floor anatomy. While TPUS can provide information comparable to other imaging modalities, its operator-dependent nature often limits its use to specialized centers; given its operator dependence, TPUS should be performed by specialists with dedicated pelvic-floor expertise (e.g., coloproctologists and urogynecologists) within a multidisciplinary setting [13]. Ultimately, the choice of imaging modality should be individualized based on patient-specific factors, resource availability, and a careful consideration of the benefits and limitations of each technique.

For patients with anterior compartment disorders or urinary incontinence, urodynamic studies and a multidisciplinary evaluation with contributions from a coloproctologist and a urogynecologist are mandatory to determine the need for concomitant surgical intervention in pelvic floor reconstruction.

Neurophysiological testing, encompassing pelvic floor electromyography, sacral reflex latency, and evoked potentials, could be considered in patients with anorectal disorders and suspected neurological involvement. These tests may help differentiate between primary bowel dysfunction and neurological etiologies [13, 15].

## Statements

*Statement 1.1:* In patients with rectal prolapse candidates for robotic ventral mesh rectopexy, a comprehensive evaluation, including physical examination, anoperineal examination, and anoscopy is recommended.

*Strong recommendation, low quality of evidence (GRADE 1 C).*

*Strength of consensus: 94%*

*Statement 1.2:* Additional diagnostic investigations, such as colonoscopy, fluoroscopic defecography, dynamic magnetic resonance defecography, anorectal manometry, anal endosonography, and urodynamic studies may be considered in cases with suspected coexisting pathologies, obstructed defecation symptoms, or impaired anal continence. These tests can also help identify associated anterior pelvic floor support defects (e.g. cystocele, vaginal vault prolapse, and enterocele).

*Strong recommendation, moderate quality of evidence (GRADE 2 C).*

*Strength of consensus: 91%*

d

**Table:** 

Question no. 2: What are the indications for robotic ventral mesh rectopexy?	

### Literature review

The available evidence on RVMR is characterized by small sample sizes, retrospective designs, and a lack of long-term follow-up, limiting the robustness of the findings. Below are the insights from the literature on various conditions:

### External rectal prolapse

Eighteen studies (325 patients) assessed RVMR outcomes for ERP [25–42], comprising case–control studies (8), case reports (5), retrospective cohort studies (3), a prospective cohort study, and a cross-sectional study. These studies highlighted consistent anatomical correction and functional improvement with low complication rates. Key findings include:

- RVMR shows comparable or improved functional outcomes (including ODS and fecal incontinence) to laparoscopic ventral mesh rectopexy (LVMR), with lower recurrence rates and better anal continence in some cases. In particular, RVMR has been associated with lower recurrence rates and higher patient satisfaction in a comparative series with short-to-mid-term follow-up (up to 3 years) [38] and with better anal continence outcomes in a multicenter matched-pair analysis with a median follow-up of 3.3 years (range 1.6–7.4) [35].
- Both procedures demonstrate high patient satisfaction and symptom improvement.
- While RVMR may involve longer operative times and higher costs, perioperative morbidity and hospital stays are similar to those of LVMR.
- Long-term outcomes for function and recurrence rates are generally favorable and comparable to LVMR, with some evidence suggesting fewer mesh-related complications with RVMR, even after extended follow-up up to 17 years [41].

Six studies (43 patients) reported outcomes of RVMR for ERP in males [29, 30, 33, 38, 43, 44]. While male-specific data are limited, findings indicate comparable anatomical and functional outcomes to females, with low complication rates and satisfactory symptom resolution.

One case–control study (73 patients) [43] and three case reports [37, 39, 40] documented RVMR for recurrent ERP, showing satisfactory anatomical correction, symptom relief, and functional outcomes comparable to primary procedures. The case–control study found no significant differences in complications or recurrence rates between primary and redo RVMR, suggesting that repeat surgery is safe and feasible. However, redo procedures are inherently complex due to potential challenges such as scarring and adhesions, requiring high surgical expertise.

### Internal rectal prolapse

Four studies (126 patients) evaluated RVMR for IRP [32, 34, 35, 45], demonstrating significant improvement in ODS and fecal incontinence, particularly in patients with concurrent rectocele. Key insights include:

- RVMR and LVMR with biological mesh appear safe and effective, showing significant symptom reduction using validated scoring systems (e.g., Cleveland Clinic Constipation Score [CCCS], Cleveland Clinic Incontinence Score [CCIS], and ODS), and high patient satisfaction at median follow-up of 3.3 years, up to 17 years in some series [35, 41].
- RVMR has gained acceptance in some institutions as a viable surgical option, with good symptom improvement, low morbidity, and low recurrence rates.
- Some evidence suggests lower mid-term anal incontinence scores with RVMR. In a multicenter matched-pair analysis with a median follow-up of 3.3 years, patients undergoing RVMR had significantly lower Wexner incontinence scores and less persistent fecal incontinence compared with LVMR [35]. However, this benefit was counterbalanced by a higher incidence of de novo pelvic pain (31.8% vs 11.8%) [35].

### Isolated rectocele and rectocele associated with enterocele among broader pelvic floor disorders

Four studies (40 patients) suggest that RVMR is feasible and effective for managing symptoms such as incomplete evacuation, pelvic discomfort, and ODS [32, 34, 41, 46]. However, evidence specifically addressing isolated rectocele is very limited, as most series included mixed pelvic floor disorders, and long-term anatomical durability remains uncertain. Key insights include:

- Both LVMR and RVMR with a biological mesh effectively reduce rectocele-related symptoms with high patient satisfaction.
- RVMR may be especially advantageous in complex or recurrent cases.

One case–control study (32 patients) comparing RVMR and LVMR for rectocele with enterocele reported greater improvement in ODS scores at a mean follow-up of 16 months, including reduced straining and the need for digital assistance, higher post-defecation satisfaction, fewer early complications, and reduced intraoperative blood loss with RVMR [27].

## Statements

*Statement 2.1:* Robotic ventral mesh rectopexy can be indicated and safely performed in patients with primary or recurrent external rectal prolapse.

*Weak recommendation, low quality of evidence (GRADE 2 C).*

*Strength of consensus: 100%*

*Statement 2.2:* Robotic ventral mesh rectopexy can be indicated and safely performed in patients with internal rectal prolapse presenting with symptoms of obstructed defecation syndrome and/or fecal incontinence, whether or not associated with other anatomical abnormalities (e.g., enterocele, rectocele), and refractory to conservative management.

*Weak recommendation, low quality of evidence (GRADE 2 C).*

*Strength of consensus: 94%*

*Statement 2.3:* Robotic ventral mesh rectopexy can be indicated and safely performed in patients with isolated rectocele or rectocele associated with enterocele after the failure of conservative management.

*Weak recommendation, low quality of evidence (GRADE 2 C).*

*Strength of consensus: 77%*

**Table:** 

Question no. 3: What is the optimal anesthetic strategy for RVMR?	

### Literature review

While no studies specifically address anesthesia management for RVMR, guidance can be drawn from the broader literature on robotic and laparoscopic abdominal surgery. Preoperative evaluation for RVMR should adhere to established guidelines for non-cardiac surgery [47–50]. Currently, no absolute contraindications to the robotic approach exist, and even patients with glaucoma generally tolerate the required positioning [51, 52]. Anesthesia teams experienced in robotic surgery should conduct preoperative assessments, considering the unique demands of this approach (robotic operating room and anesthesia console layout, patient positioning, and planning of vascular access due to the challenges of patient accessibility during surgery) [47, 53].

Analgesia for RVMR should prioritize early mobilization and oral intake while minimizing opioid-related side effects. A multimodal approach, incorporating peripheral nerve blocks and NSAIDs, is recommended within an Enhanced Recovery After Surgery (ERAS) framework [54]. Ultrasound-guided transversus abdominis plane (TAP) blocks have proven particularly effective in reducing opioid consumption and hastening bowel recovery [55, 56]. Intrathecal morphine, while effective, carries a higher risk of side effects and should be avoided in favor of alternative strategies [54, 57, 58]. Perioperative lidocaine and ketamine infusions have shown promise in reducing opioid requirements and postoperative nausea and vomiting [59, 60].

Fluid management for RVMR should adhere to ERAS guidelines, including limited fasting and carbohydrate loading. Intraoperatively, balanced crystalloids should be preferred over colloids, which should be reserved for significant acute volume loss [54, 61]. A neutral fluid balance is generally recommended, with goal-directed fluid therapy utilizing stroke volume monitoring for high-risk patients. Excessive fluid administration should be avoided to minimize the risk of pulmonary complications, ileus, and delayed recovery [54, 62–64].

## Statements

*Statement 3.1:* Preoperative evaluation for patients undergoing robotic ventral mesh rectopexy should be conducted by anesthesiologists following the current guidelines for the preoperative assessment of non-cardiac surgery patients.

*Strong recommendation, moderate quality of evidence (GRADE 1B).*

*Strength of consensus: 100%*

*Statement 3.2:* For analgesia during robotic ventral mesh rectopexy, opioid-sparing protocols are recommended. These may include intraoperative lidocaine and ketamine infusions, as well as the use of a transversus abdominis plane block. The use of intrathecal morphine should be avoided due to its associated side effects.

*Weak recommendation, moderate quality of evidence (GRADE 2B).*

*Strength of consensus: 88%*

*Statement 3.3:* During the perioperative period, balanced crystalloid solutions are recommended, while normal saline should be avoided. The use of colloids should be minimized and limited for cases of severe hemodynamic instability due to volume loss. The goal should be achieving a near-zero fluid balance. In high-risk patients, advanced hemodynamic monitoring and goal-directed fluid therapy may be considered.

*Weak recommendation, moderate quality of evidence (GRADE 2B).*

*Strength of consensus: 83%*

**Table:** 

Question no. 4: What training/experience should the surgeon have before performing robotic ventral mesh rectopexy? Is the learning curve for robotic ventral mesh rectopexy shorter compared to the learning curve for laparoscopic ventral mesh rectopexy?	

### Literature review

The learning curve for LVMR was defined by Mackenzie and Dixon in 2014 through a CUSUM analysis performed on 636 LVMR cases [65]. A change point for operative time was detected after 54 cases, for recurrence after 82 cases, for postoperative complications after 87 cases, and for hospital stay after 88 cases. The authors also considered functional outcomes, noting a change in the CCIS after 105 cases, in the Birmingham Bowel and Urinary Symptoms Questionnaire-22 (BBUSQ-22) at 3 months after 87 cases, and the BBUSQ-22 at 1 year after 91 cases. This article set the benchmark in the scientific literature for LVMR.

In 2013, Perrenot et al. were the first to define the learning curve for RVMR [66]. The learning curve for operative time, calculated using a CUSUM analysis, revealed a turning point after 18 patients. This curve was derived from a single surgeon performing most of the procedures in the reported series. However, this study was limited by selection bias due to variations in surgical technique (rectopexy with one ventral mesh, rectopexy with two anterolateral meshes, and suture rectopexy with or without sigmoid resection). More recently, the learning curve for RVMR was analyzed by Chaoui et al., based solely on operative time, with the learning phase and competency phase reached after 9 and 22 cases, while the mastery phase started after 23 cases [67].

Other studies have compared RVMR and LVMR learning curves. Mäkelä-Kaikkonen et al. found no significant difference in operative time between the two approaches, although a progressive reduction was observed throughout their series [68]. Dumas et al. reported that robotic operative time reached laparoscopic levels after 15 cases, but differences in surgical technique and timing of procedures limited their results [38]. Conversely, van der Schans et al. identified turning points for operative time after 36 and 55 cases for two surgeons, respectively, with no differences in complication rates, confirming the safety of the robotic procedures even during the learning phase [69].

These discrepancies highlight the influence of surgeon experience, prior robotic expertise, patient characteristics, and specific surgical techniques on the RVMR and LVMR learning curves. Furthermore, these reports often include a small proportion of RVMR cases, limiting their generalizability. Advancements in technology, such as telementoring programs, may offer new opportunities for structured RVMR training. Butt et al. recently reported a plateau phase for RVMR after 12 cases in a telementoring program [70]. The introduction of new robotic platforms will likely present further challenges in defining the RVMR learning curve [71–74].

Despite widespread assumptions about skill translation in surgery, direct comparative evidence specifically assessing the transferability of LVMR skills to RVMR is not available. Much of the learning-curve literature in this area is designed to capture the early adoption phase and, to minimize confounding, commonly enrolls surgeons at or near their first robotic cases. By construction, such designs either exclude substantial prior robotic proficiency or do not stratify outcomes by pre-existing robotic experience. As a result, the available studies on RVMR estimate the robot-specific learning curve rather than the incremental benefit of prior laparoscopic expertise. In some series, participating surgeons are explicitly described as having no prior robotic experience (while often being experienced laparoscopic rectopexy operators), which maximizes internal validity for curve estimation but precludes quantification of true skill transfer from laparoscopy to robotics in RVMR [69]. Because RVMR-specific transfer studies are lacking, insight must be drawn from broader research on laparoscopy-to-robotics skill transfer. Systematic reviews and meta-analyses across specialties show a mixed but frequently positive transfer effect from laparoscopy to robotics, especially for advanced psychomotor tasks such as intracorporeal suturing and knot-tying [75, 76]. By contrast, open-to-robotic transfer shows little consistent benefit [77]. Several reviews conclude that prior laparoscopic training can enhance early robotic performance in simulated and clinical contexts, though heterogeneity in tasks, assessment tools, and trainee baselines determined conflicting results, preventing definitive conclusions [75–77]. Taken together, this literature supports plausible positive transfer for laparoscopic surgeons adopting robotics, notably in needle handling, economy of motion, and depth perception strategies, even if the magnitude of that transfer cannot be quantified for RVMR under current evidence. It is also pertinent that a strand of learning-curve research deliberately moves in the opposite direction of what would be needed to study skill transfer: to avoid bias, investigators often prefer novices to the robotic platform, or at least cohorts with standardized minimal robotic exposure, precisely to prevent prior experience from masking the curve. While methodologically sound for defining curve length, this approach necessarily limits inferences about how much pre-existing laparoscopic competence abbreviates the robotic learning pathway in RVMR. Finally, the question of cross-platform transferability between different robotic systems remains largely unexplored in RVMR. Early implementation reports and set-up standardization papers with newer platforms emphasize platform-specific workflows (port maps, arm kinematics, bedside assistance), reinforcing that competencies do not translate in a strictly one-to-one fashion across systems and that platform-tailored orientation and credentialing are advisable [78]. As multiple systems enter practice, future studies should stratify learning metrics by platform and report whether previously acquired robotic proficiency shortens the adoption curve when switching systems.

## Statements

*Statement 4.1:* Structured training following national regulations could be suggested to perform robotic ventral mesh rectopexy. However, no formal definition of robotic ventral mesh rectopexy training currently exists, and further studies are required to ensure the safe adoption of this surgical technique for novice surgeons.

*Weak recommendation, very low quality of evidence (GRADE 2D).*

*Strength of consensus: 91%*

*Statement 4.2:* A potentially shorter learning curve is associated with robotic ventral mesh rectopexy compared to laparoscopic ventral mesh rectopexy, but the precise threshold cannot be determined due to the limited evidence.

*Weak recommendation, low quality of evidence (GRADE 2 C).*

*Strength of consensus: 86%*

*Statement 4.3:* The learning curve for robotic ventral mesh rectopexy should be evaluated considering technical and non-technical skills, previous expertise, availability of a structured program, access to the robotic platform, and caseload.

*Weak recommendation, low quality of evidence (GRADE 2 C).*

*Strength of consensus: 91%*

**Table:** 

Question no. 5: For robotic ventral mesh rectopexy, what is the recommended setup regarding the positioning of the trocars, the docking of the robot, and the surgical instruments necessary for the procedure?	

### Literature review

For the past 25 years, the da Vinci Surgical System (Intuitive Surgical Inc., Sunnyvale, CA, USA) has been the leading platform for robotic-assisted surgery, and it has significantly contributed to the standardization and reproducibility of numerous surgical procedures [79, 80]. Since 2022, the emergence of new robotic platforms has diversified the robotic surgery landscape, introduced novel technical concepts, and provided alternatives in surgical techniques, thereby challenging the notion of a single standardized approach to robotic surgery [74, 81]. Hence, the current literature on RVR mainly focused on the da Vinci Surgical System.

**Fig. 1:**
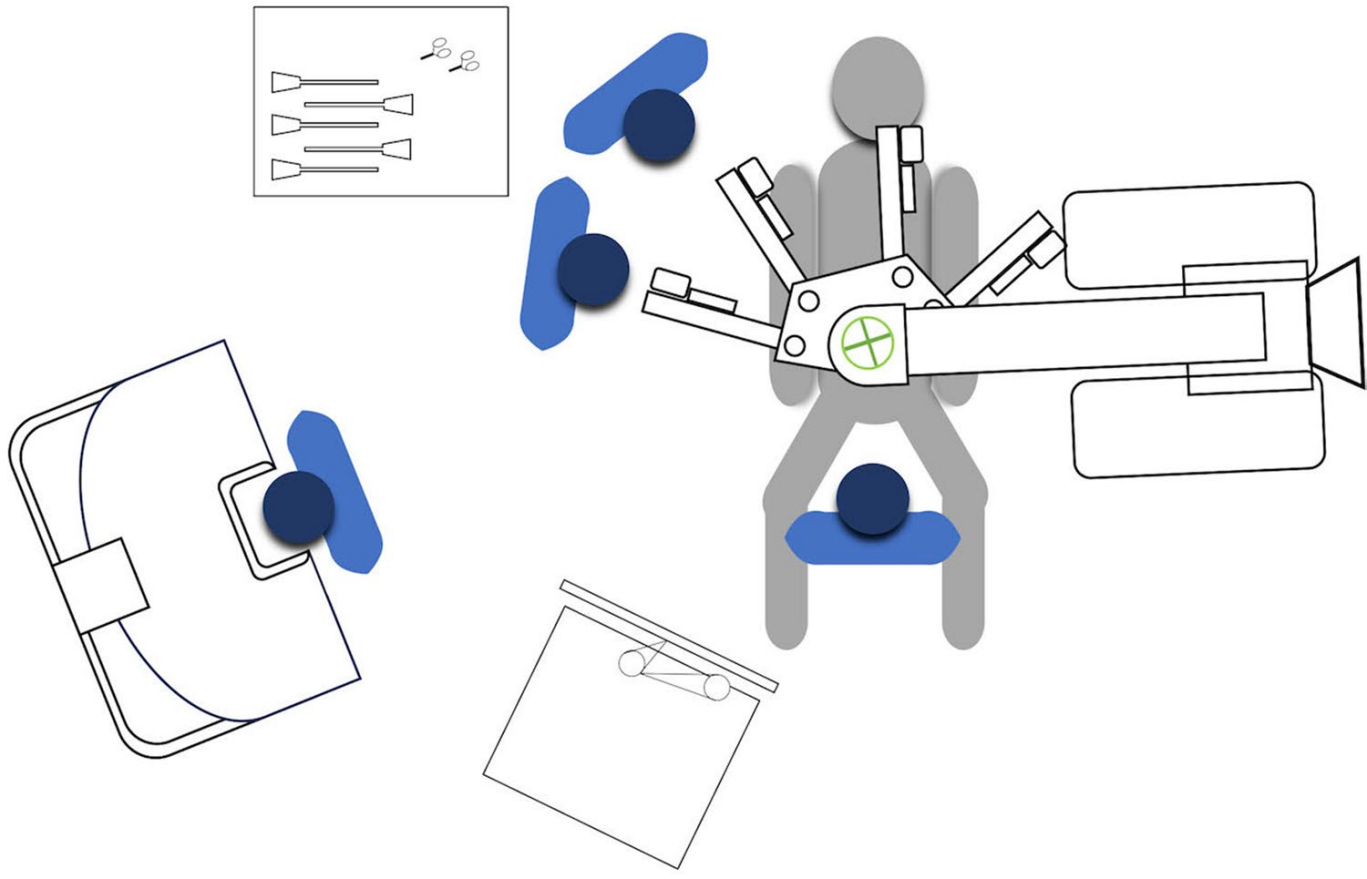
Operating room layout and team setup for robotic ventral mesh rectopexy (RVMR)

The patient is placed in a lithotomy position with steep Trendelenburg and a right tilt to facilitate gravitational displacement of the small bowel and adequate pelvic exposure. No well-powered, large-scale studies have been published comparing different trocar placement, patient cart positioning, and robotic instrument selection in RVR. The typical port configuration for the da Vinci multi-arm (X/Xi) Surgical System involves four 8-mm robotic ports and one 5-mm or 12-mm assistant port, arranged in a straight line or a semi-arcuate configuration [1, 38, 68, 82, 83]. The patient cart is docked from the patient’s left side (targeting is done towards the pelvis), with the assistant surgeon and scrub nurse standing on the patient’s right side. Figure 1 illustrates a representative operating room layout and team setup for RVMR. The standard configuration employs the following EndoWrist® instruments: a) monopolar cautery (monopolar curved scissors or permanent cautery hook) or needle drivers on arm 4; b) bipolar instruments on arm 2; c) graspers (ProGrasp forceps or Tip-Up fenestrated grasper) on arm 1. Recently, Marra et al. proposed some technical modifications to optimize robot-related costs in RVMR [44]. First, robotic arms and ports were reduced from the traditional four to three, while laparoscopic assistance was strengthened through two ports. Second, the selection of robotic instruments was limited to Cadiere forceps, monopolar curved scissors, and a large needle driver. Despite the limited sample size, the authors reported a significant reduction in the overall cost of hospitalization (6604.5 ± 589.5 vs. 8755.0 ± 906.4 €) and operating room time (201 ± 26 vs. 253 ± 16 min). The current literature lacks consistent data on Hugo™ RAS system [71, 72] (only 2 case reports), CMR Versius system [78] (only 1 case report), and da Vinci SP [40] (only 1 case report).

## Statements

*Statement 5.1:* For the da Vinci multi-arm (X/Xi) Surgical System, common trocar positioning requires four robotic ports and one assistant port, often in a straight or semi-arcuate configuration. The patient cart is usually docked from the patient's left side. Alternative robotic setups can be employed according to the surgeon’s preferences, local resource availability, and hospital economic strategy.

*Expert opinion.*

*Strength of consensus: 91%*

*Statement 5.2:* For the da Vinci multi-arm (X/Xi) Surgical System, common robotic surgical instruments employed for robotic ventral mesh rectopexy include EndoWrist monopolar cautery instruments, needle drivers, bipolar instruments, and graspers. The choice of robotic surgical instruments typically relies on the surgeon's preferences, local resource availability, and hospital economic strategy.

*Expert opinion.*

*Strength of consensus: 94%*

*Statement 5.3:* There is insufficient data to recommend a specific robotic setup for robotic platforms other than the da Vinci X/Xi Surgical System.

*Expert opinion.*

*Strength of consensus: 88%*

**Table:** 

Question no. 6: For robotic ventral mesh rectopexy, should a prosthetic mesh reinforcement always be recommended? What are the optimal prosthetic materials and fixation techniques for robotic ventral mesh rectopexy?	

### Literature review

#### The use of mesh

The role of mesh in rectopexy procedures has been evaluated in three RCTs [84–86]. Lundby et al. compared LVMR using polypropylene mesh to laparoscopic posterior suture rectopexy in 75 patients, finding no significant difference in the improvement of ODS scores and recurrence within 12 months (0% in the mesh group vs. 5% in the suture rectopexy group, p = 0.305) [85]. Luukkonen et al. compared posterior mesh rectopexy with polyglycolic acid mesh to posterior suture rectopexy combined with sigmoidectomy in 30 patients, noting that both techniques effectively controlled RP with no significant differences in complications or functional outcomes [86]. Emile et al. compared LVMR using polypropylene mesh to Delorme's perineal procedure in 50 patients, finding no significant differences in recurrence rates (8% vs. 16%) or symptom improvement, although the mesh group had a longer operative time, but shorter hospital stay [84]. Across these studies, mesh use was associated with a lower, though statistically insignificant, recurrence rate, although limited by small sample sizes and variability in control interventions.

However, broader studies addressing both IRP and ERP suggest favorable long-term cost-effectiveness for mesh-based techniques, particularly with minimally invasive approaches like LVMR and RVMR, which are associated with shorter hospital stays and low complication rates [2, 87, 88]. Mesh use appears to offer benefits in anatomical restoration and recurrence prevention. Studies indicate that mesh fixation, whether synthetic or biological, contributes to durable outcomes by stabilizing the rectum and minimizing the need for extensive dissection, thereby reducing the risk of autonomic nerve damage and postoperative bowel dysfunction [89, 90]. Furthermore, in comparing mesh types, non-absorbable materials like polypropylene have shown lower, though not statistically significant, recurrence rates compared to absorbable or biological meshes in short-term follow-up [90, 91]. Although mesh does not consistently outperform non-mesh techniques in functional outcomes, its potential durability and ability to reduce recurrence make it a valuable option for appropriately selected patients undergoing RVMR.While ventral rectopexy is generally effective in treating RP and associated symptoms like fecal incontinence and ODS, it is crucial to recognize the potential for new-onset or worsening constipation and ODS postoperatively. A study by Mäkelä-Kaikkonen et al. comparing RVMR and LVMR found that both methods were safe and effective, but one patient in each group experienced worsened ODS symptoms post-surgery [68]. Similarly, Portier et al. demonstrated the efficacy of ventral rectopexy in treating fecal incontinence associated with internal rectal intussusception but also reported new-onset constipation in a small proportion of patients [92]. Tsunoda et al. confirmed the anatomical correction achieved through LVMR for recto-anal intussusception but observed de novo recto-rectal intussusception or persistent ODS in some patients, highlighting that anatomical correction does not always guarantee functional improvement [93]. This potential for new or worsened constipation and ODS can be attributed to several factors, including nerve disruption during surgery and altered rectal function due to mesh implantation. Therefore, thorough preoperative counseling is essential to manage patient expectations and ensure informed decision-making. Patients should be explicitly informed about the possibility of new or persistent constipation and ODS following ventral rectopexy, regardless of the surgical approach (robotic or laparoscopic).

Beyond disease recurrence and ODS, harms reported after VMR include erosion/exposure and fistula formation, chronic pelvic or neuropathic pain, dyspareunia/sexual dysfunction, deep prosthesis-related infection, and the rare but severe entity of sacral spondylodiscitis/osteomyelitis following promontory fixation. Additional risks include mesh detachment/migration, adhesive complications (including obstruction), re-operation, and psychosocial burden. These events are uncommon but potentially life-altering and should be discussed in a balanced manner, together with the possibility of persistent or de novo ODS symptoms despite anatomical correction.

The UK Independent Medicines and Medical Devices Safety Review (the Cumberlege Review, “First Do No Harm”, 2020) [94–96] examined systemic responses to patient-reported harms from medicines and devices, including pelvic mesh. Two themes are directly relevant to RVMR: 1) listen to patients and treat their reports as safety signals, and 2) hard-wire governance through independent oversight, specialist centres, comprehensive data registries, and clearer consent and redress pathways. The Review issued nine recommendations, among them the creation of a Patient Safety Commissioner to champion the patient voice; establishment of specialist referral services for mesh-related harm; stronger registries and device traceability; reforms to consent, candor, and complaints; and a move toward non-adversarial redress for avoidable harm. Post-Cumberlege implementation in the UK includes specialized multidisciplinary centres commissioned to manage mesh complications across regions, with teams spanning surgery, imaging, nursing, physiotherapy, pain, and psychology—an operational model that can inform analogous services for rectopexy complications. Centres should maintain links with national registries and publish outcomes to support transparent, data-driven improvement. While framed for England, these principles align with our consensus and offer a transferable blueprint for service design. In practical terms, for RVMR, this translates into pre-operative multidisciplinary triage, documentation of device specifics, routine registry entry, explicit discussion of both known and unknown risks and complications, clear referral routes to designated services if complications occur, and structured written consents (documenting the indication for surgery and alternatives, the proposed mesh type and rationale, and the full spectrum of potential mesh- and procedure-related complications).

### Prosthetic materials and fixation techniques

A systematic review by Hess et al. provided a comprehensive analysis of mesh-related complications in LVMR and RVMR [97]. This review, encompassing 40 studies (3 RCTs, 13 prospective, and 24 retrospective studies) and 6,269 patients, found that while ventral mesh rectopexy is generally safe, complications occur in approximately 9.2% of cases, with mesh-related complications accounting for 1.4%. Erosion was the most frequent mesh-related complication (64.8%), occurring more often with synthetic meshes. Synthetic meshes, while durable, were associated with a higher risk of erosion and fistula formation compared to biological meshes [97–99]. Multiple series indicate that polyester prostheses are associated with a higher probability of mesh exposure and erosion than polypropylene implants. In a large multicenter international cohort, polyester showed a markedly increased hazard of erosion compared to polypropylene (HR 4.09, 95%CI 2.16–7.73) [99]. Accordingly, polyester mesh should be avoided in ventral rectopexy, as it is associated with increased morbidity [82, 100, 101]. When a synthetic prosthesis is selected, lightweight, macroporous polypropylene is generally considered the most favorable option, with biologic grafts reserved for selected clinical scenarios, alongside registry participation and structured follow-up. Biological meshes, although used less frequently, demonstrated a lower complication rate but might be associated with higher long-term recurrence rates [97, 98]. Mesh fixation techniques also influenced outcomes [97]. Non-absorbable sutures were the most common fixation method, valued for their durability. Combining absorbable and non-absorbable sutures resulted in comparable complication rates to non-absorbable sutures alone. Fixation methods typically involved sutures, tackers, or a combination, with single-method fixation appearing slightly superior, particularly with synthetic meshes. The highest complication rate (2%) was observed in a group with a non-specified mesh type using suture-only fixation. The review emphasized the importance of preventing mesh release, a rare but serious complication, which was less common with biological meshes, likely due to their resorbable nature [97]. These findings underscore the importance of carefully considering mesh type and fixation technique in RVMR. The choice of surgical technique and mesh material is often influenced by the surgeon’s expertise and patient-specific factors, including comorbidities and the presence of concomitant pelvic floor disorders [2, 68]. Titanized meshes are typically composed of polypropylene with a titanium coating, offering superior biocompatibility and potentially reducing the risk of inflammation and mesh-related complications. In a study evaluating the outcomes of a modified robotic ventral rectopexy with a folded single titanized polypropylene mesh, 22 women with complex pelvic organ prolapse were treated and followed for 12 months. The procedure demonstrated a significant improvement in both anatomical and functional outcomes. Indeed, a complete resolution of bulging symptoms was observed in 95.4% of patients, with notable improvements in Pelvic Organ Prolapse Quantification (POP-Q) and Wexner constipation scores. No mesh-related complications, such as erosion or new-onset dyspareunia, were reported at 12 months [102].

Shepherd et al. investigated the impact of suture type on mesh/suture complications in sacrocolpopexy using polypropylene mesh [103]. They found a significantly higher erosion rate with polyester (Ethibond) sutures (3.7%) compared to polydioxanone sulfate (PDS) sutures (0%). In the context of LVMR, a consensus statement by Mercer-Jones et al. recommended the use of PDS sutures for vaginal fixation with any mesh type and for rectal fixation when using synthetic mesh [104].

## Statements

*Statement 6.1:* The use of a prosthetic mesh for abdominal rectopexy may reduce the risk of recurrence and can be considered in patients with full-thickness rectal prolapse or posterior pelvic floor disorders. However, it is crucial to inform and adequately counsel patients about the potential risks, such as de novo constipation or obstructive defecation syndrome.

*Weak recommendation, low quality of evidence (GRADE 2 C).*

*Strength of consensus: 97%*

*Statement 6.2:* Both biological and synthetic meshes can be considered for robotic ventral mesh rectopexy. Synthetic meshes generally offer lower recurrence rates but are associated with higher risks of fistulation and erosions.

*Weak recommendation, very low quality of evidence (2D).*

*Strength of consensus: 88%*

**Table:** 

Question no. 7: What are the surgical steps to be followed for robotic ventral mesh rectopexy?	

### Literature review

The ventral mesh rectopexy technique, initially described by D'Hoore and Penninckx in 2004 [105], has progressively gained global acceptance for the surgical correction of rectal and pelvic organ prolapse [1, 16, 82, 101, 106–108], becoming the standard of care for RP in Europe [15, 18, 106, 109, 110]. The main characteristic of this procedure is that the dissection is confined to the anterior aspect of the rectum, minimizing the risk of injury to posterolateral structures, including autonomic nerves. This contrasts with the Orr-Loygue procedure, which entails extensive anterior and posterior rectal dissection to the level of the levator ani muscles (including excision of the Douglas pouch) and involves fixing two meshes to the anterolateral rectal walls and the sacral promontory [111]. Nowadays, RVR is performed according to the laparoscopic technique described by D’Hoore et al. [105]. In detail, the first surgical step consists of exposing the pelvis by retracting the small bowel and sigmoid colon away from the pelvic cavity. The sigmoid colon is retracted anteriorly, cranially, and laterally, then the peritoneal incision starts at the base of the rectosigmoid mesentery, medially to the right common iliac artery, identifying the avascular areolar plane along the sacral promontory and exposing the presacral fascia. The peritoneal incision is continued on the right side of the rectum down to the pouch of Douglas in an inverted J-form (the right hypogastric nerve plexus and ureter can be identified and preserved during this surgical step). The dissection is then extended through Denonvillier’s fascia along the anterior mesorectal surface, opening the rectovaginal space in females and rectovesical space in men, down to the levator ani plane. D’Hoore initially described the fixation of the mesh to the ventral aspect of the low rectum, posterior vaginal fornix, and sacral promontory (fixation techniques for RVR are described in question number 6). The peritoneal incision is completely closed to prevent mesh exposure using absorbable barbed running sutures.

Several variations of the D’Hoore technique have subsequently been described, for instance, without performing posterior colpopexy [82] or anchoring the mesh to the levator muscles anterior to the rectum (levatorpexy) and not directly to the rectum [112, 113]. The inverted J-form peritoneal incision can be realized in two steps: the first peritoneal incision can be developed along the medial part of the right uterosacral ligament without reaching the rectovaginal space; then a second independent peritoneal incision opens the rectovaginal space and clears the anterior rectal wall, thus rejoining the first one [114]. Moreover, the creation of a retroperitoneal tunnel via limited peritoneal incisions at the Douglas pouch apex and sacral promontory (replacing the traditional inverted J incision) has been described as an alternative approach for RVR [44, 115]. Alternatively, Fraccalvieri et al. described the longitudinal plication of the anterior rectal wall (anterior rectoplasty) before mesh fixation [116]. Direct comparative data supporting routine fixation of the sigmoid colon as an adjunct to ventral rectopexy are lacking, and fixation may plausibly increase segmental fixity and reduce rectosigmoid compliance above the pexy, thereby predisposing to de novo outlet obstruction [117]. Contemporary prognostic analyses indicate that redundant sigmoid colon and pre-existing constipation are associated with a higher likelihood of persistent or new-onset constipation after ventral mesh rectopexy, suggesting that when redundancy is clinically relevant, resection rectopexy, rather than added fixation, may be the more appropriate strategy for constipation control, as supported by comparative studies and guideline summaries (noting that resection rectopexy lies outside the scope of this consensus, which addresses RVMR only) [16, 86, 118].

In 2022, the International Robotic Rectopexy Delphi Group recognized that the dissection down to the pelvic floor (100% agreement), rectovaginal septum dissection (85% agreement), and placement of the mesh (90% agreement) are the most important technical steps when performing RVR, according to the panel of twenty surgeons [119].

## Statements

*Statement 7.1:* The autonomic nerve-sparing ventral mesh rectopexy described by D’Hoore represents the reference technique also in case of robotic approach, but technical variations (e.g., without posterior colpopexy, levatorpexy) could be considered according to imaging, clinical evaluation, symptoms, disease characteristics, and patient’s centered outcomes.

*Weak recommendation based on low-quality evidence (GRADE 2 C).*

*Strength of consensus: 100%*

*Statement 7.2:* The peritoneum should be incised on the right side of the rectum starting at the level of sacral promontory, medially to the right common iliac artery, down to the pouch of Douglas in an inverted J-form and preserving the homolateral hypogastric nerve plexus and ureter. The dissection should be limited to the anterior rectum and remain superficial, minimizing the risk of potential complications of a posterior rectal dissection.

*Expert opinion.*

*Strength of consensus: 97%*

*Statement 7.3:* The presacral fascia should be exposed on its medial-right side. During the dissection of the sacral promontory, caution should be paid to avoid presacral nerve plexus damage.

*Expert opinion.*

*Strength of consensus: 100%*

*Statement 7.4:* The complete ventral (anterior) dissection of the rectovaginal space in females and rectovesical space in men is a crucial surgical step in robotic ventral mesh rectopexy. The dissection should be developed down to the perineal body as distally as possible. After securing the mesh in place, the peritoneum is closed using a continuous absorbable suture covering the mesh along its entire length.

*Expert opinion.*

*Strength of consensus: 94%*

d

**Table:** 

Question no. 8: What type of surgical procedure could be combined in case of multicompartment prolapses during robotic ventral mesh rectopexy?	

### Literature review

Combined surgical intervention for POP and RP is gaining interest, as concomitant prolapse could be more prevalent than isolated compartment defects. Up to half of RP patients present with POP symptoms, and POP is frequently associated with ODS and IRP [120]. For these patients, a combined surgical approach, with multidisciplinary collaboration among urologists, gynecologists, and colorectal surgeons, offers significant symptom relief and quality-of-life benefits [121]. Advantages include reduced anesthesia exposure, a single hospital stay and recovery period, and decreased time off work [122], without increased operative risk [123].

Robotic surgery, despite its cost, in procedures like multicompartmental POP repair may offer technical advantages over standard laparoscopy, including enhanced dexterity, improved visualization, and superior ergonomics, potentially facilitating complex surgical maneuvers and potentially improving outcomes [124, 125]. Nevertheless, current evidence regarding the benefits of robotic assistance in combined POP repair is limited. While robotic surgery has gained popularity in various surgical fields, there is insufficient data to conclusively demonstrate its superiority over traditional approaches in terms of ease, effectiveness, safety, and reproducibility for combined POP reconstruction.

Minimally invasive sacrocolpopexy is considered a reference technique for multicompartmental POP, demonstrating good anatomical and functional outcomes [126–129]. For patients with symptomatic vaginal vault prolapse, cystocele, rectocele, and enterocele, combined RVMR with robotic sacrocolpopexy has shown promising results. This approach is associated with increased efficacy, low complication rates, and shorter hospital stays [121–124, 126–129]. Technically, RVMR is performed first, followed by dissection of the vesico-vaginal space. A Y-shaped mesh is then anchored to the vaginal wall and suspended to the sacral promontory. Careful dissection and planning for mesh fixation (e.g., separate mesh fixation) are crucial to ensure proper tension in each compartment [125, 130]. Reddy et al. reported the first successful series of combined robotic sacrocolpopexy and RVMR in 10 patients [131]. More recently, Devane et al. published a larger retrospective study of 281 patients, demonstrating feasibility, low morbidity, and short hospital stays [132]. Wallace et al. found similar complication and recurrence rates between combined POP and RP surgery and POP-only procedures, although their study was limited by treatment heterogeneity [133]. Gee et al. highlighted the safety, high patient satisfaction, and symptom improvement (enhanced defecatory function, sexual health, and overall quality of life) associated with combined sacrocolpopexy and RP repair [134]. Campagna et al. (2023) described the first robotic sacrocolpopexy plus RP repair using the Hugo RAS system, but further research is needed to evaluate this novel robotic platform [72].

Emerging combined techniques, such as robotic lateral colposuspension [135] combined with RVMR, warrant further investigation for managing advanced anterior and apical prolapse (cystocele with hysterocele or vaginal vault prolapse) with concomitant RP.

Ultimately, the decision for a combined robotic approach to multicompartmental POP should be individualized through multidisciplinary collaboration and patient-centered discussions [136].

## Statements

*Statement 8.1:* For multicompartmental prolapses, robotic ventral mesh rectopexy could be combined with other pelvic organ prolapse reconstructive procedures (e.g. sacrocolpopexy) according to the surgeon’s experience, imaging, and patient characteristics.

*Weak recommendation, low quality of evidence (GRADE 2 C).*

*Strength of consensus: 88%*

*Statement 8.2:* The decision to perform a combined robotic approach for multicompartmental pelvic organ prolapse must be taken by a multidisciplinary team.

*Weak recommendation, low quality of evidence (GRADE 2 C).*

*Strength of consensus: 88%*

**Table:** 

Question no. 9: Does the robotic approach for rectopexy provide short and/or long-term advantages compared with laparoscopy?	

### Literature review

A limited number of studies have directly compared LVMR and RVMR. The only RCT on this topic, conducted in Finland by Mäkelä-Kaikkonen et al., compared 30 patients randomly assigned to LVMR or RVMR for total RP or intussusception with ODS and/or fecal incontinence [88, 137]. This study found that RVMR was safe and effective, with similar short-term outcomes to LVMR in terms of anatomical changes (measured by POP-Q), functional improvements, and complication rates. An intermediate (24-month) follow-up of this RCT confirmed comparable results for health-related quality of life (HRQoL), anatomical correction, and functional outcomes [87]. Furthermore, an economic analysis also suggested that RVMR, despite higher initial costs, may be more cost-effective in the long term (2 and 5 years). Five-year follow-up of this RCT demonstrated sustained anatomical correction and a potential advantage for RVMR in symptom-specific quality-of-life measures, such as the Pelvic Floor Distress Inventory (PFDI-20), subscales of pelvic organ prolapse (POPDI-6), and Colorectal–Anal Distress Inventory (CRADI-8) [138]. A more recent retrospective multicenter matched-paired analysis by Laitakari et al., partially including the aforementioned RCT population, compared 152 RVMR patients to 152 LVMR patients [35]. This study found no difference in overall quality of life but reported lower postoperative Wexner Incontinence Scores, fewer ongoing incontinence symptoms, and less postoperative fecal incontinence discomfort in the RVMR group after a median follow-up of 3.3 years. RVMR patients also had shorter hospital stays but experienced more frequent de novo pelvic pain [35].

Beyond the aforementioned RCT, evidence comparing LVMR and RVMR is primarily based on non-randomized studies with varying methodologies and patient populations.

Mantoo et al. reported a case–control study comparing 44 RVMR patients to 74 historical LVMR patients with multicompartment pelvic floor dysfunctions, finding longer operative times, less blood loss, fewer early complications, and acceptable early recurrence rates with RVMR [27]. Functional outcomes, including ODS scores, also favored RVMR [27]. Wong et al. analyzed 63 patients with complex rectocele, finding longer operative times but lower blood loss with RVMR, and no difference in conversion rates, hospitalization duration, or 6-month recurrence rates [112]. More recently, Drissi et al. reported a retrospective case series of 269 patients, including 47 who underwent RVMR [41]. While RVMR was associated with a shorter length of stay, no differences were found in complication rates, functional outcomes, or recurrence rates at a median follow-up of 14 months. However, the groups differed in terms of concomitant anterior fixation and mesh type, potentially confounding the results. Heemskerk et al. published two reports from the same institution comparing LVMR and RVMR for full-thickness RP [25, 139]. While the first report found comparable complication rates but higher operative times and costs with RVMR, the second report suggested higher recurrence rates with RVMR compared with open surgery. However, both studies included techniques different than ventral rectopexy and lacked information on the surgeon's learning curve, limiting the interpretation of their findings.

Other retrospective studies have reported conflicting results. Buchs et al. found no differences in short-term outcomes between LVMR and RVMR in a small cohort of 5 patients per group [26]. Mehmood et al. observed longer operative times but better Wexner scoring, Fecal Incontinence Severity Index (FISI), and SF-36 questionnaires with RVMR in 51 patients with ERP [28]. Faucheron et al. reported comparable outcomes between LVMR and RVMR, except for reduced postoperative pain with RVMR, in 20 patients undergoing day-case surgery [29]. Brunner et al. (2017) found similar surgical and functional outcomes between LVMR and RVMR in 123 patients with descending perineum, rectocele, enterocele, intussusception, full-thickness RP, or a combination of the previous disorders [32]. Wlodarczyk et al. reported higher costs and longer operative times with RVMR but no differences in clinical outcomes in 52 patients [140]. More recently, Dumas et al. observed a lower conversion rate but longer operative time with RVMR, and similar postoperative outcomes except for a shorter length of stay and potentially better functional results at short-term follow-up [38]. In 2024, Chaoui et al. reported no difference in operative time or functional outcomes between LVMR and RVMR in 149 patients, but RVMR was associated with a shorter length of stay and higher costs [67].

Overall, while the RCT by Mäkelä-Kaikkonen et al. provides the highest level of evidence, the limited number of comparative studies and the heterogeneity of non-randomized studies make it challenging to draw definitive conclusions about the relative merits of LVMR and RVMR. Further high-quality research is needed to clarify the role of robotics in ventral mesh rectopexy and identify patient subgroups who may benefit most from this approach.

## Statements

*Statement 9.1:* Robotic and laparoscopic ventral mesh rectopexy showed comparable clinical outcomes and can be considered alternative techniques. Robotic ventral mesh rectopexy may offer some benefits in long-term quality-of-life outcomes.

*Weak recommendation, low quality of evidence (GRADE 2 C).*

*Strength of consensus: 82%*

*Statement 9.2:* Robotic ventral mesh rectopexy incurs higher overall costs compared to laparoscopic ventral mesh rectopexy. Cost-effectiveness should be evaluated in a specific context and include long-term data, considering surgical expertise, team preparation, national regulations, pricing policies, and reimbursement policies.

*Weak recommendation, low quality of evidence (GRADE 2 C).*

*Strength of consensus: 86%*

**Table:** 

Question no. 10: What types of studies and research should be encouraged in the field of robotic surgery for rectal prolapse?	

### Literature review

While robotic surgery for RP appears promising, the current literature is limited, consisting mainly of small retrospective studies and case series with short-term follow-up. The only published RCT comparing RVMR and LVMR for RP was conducted in Finland in 2012 [87, 137, 138]. Another study by Mehmood et al., initially described as an RCT, was later acknowledged as a non-randomized study due to the lack of a proper randomization procedure [28]. These studies suggest that robotic surgery offers potential advantages, such as improved ergonomics, precise dissection in confined spaces, and comparable outcomes to laparoscopy in terms of recurrence and complications, with potential benefits in hospital stay and quality of life. However, long-term data and high-quality evidence remain limited.

To definitively establish the role of robotic surgery in RP, further high-quality research is needed. Future studies should focus on long-term functional outcomes (> 5 years), RVMR learning curve, patient-centered outcomes (pain, comfort, satisfaction), patient preferences, cost-effectiveness, and comparisons between different robotic platforms [74]. These studies will help define standardized techniques and clarify the optimal approach for RP management.

## Statements

*Statement 10.1:* Key Research Priorities – Outcome Measures:

- Clinical outcomes, such as functional outcomes, recovery time, patient-centered outcomes (e.g., pain, comfort, satisfaction), and quality of life.
- Cost-effectiveness and risks/benefits evaluation.
- Safety and delayed adverse events, recurrence rates, and secondary interventions.
- Patients’ preferences evaluated before the intervention, to integrate this information into the decision-making process, based on the principles of evidence-based medicine.

Weak recommendation, low quality of evidence (GRADE 2 C).

Strength of consensus: 97%

*Statement 10.2:* Key Research Priorities – methodological considerations:

- Multi-center prospective cohort studies and study registries to address clinical and patient-reported outcomes according to the institutional case volume and surgeon’s experience.
- Prospective studies, observational, and RCTs to assess long-term (> 5 years) outcomes and monitor results, stability/recurrence, over time.
- In the case of observational and non-randomized studies, controlling the confounding via the propensity score approach or multivariable models is recommended.
- Advanced big data analytical techniques, including data modeling and machine learning approaches, can improve patient profiling outcomes in scenarios where traditional randomization is challenging or impractical.
- Patient-reported outcomes and functional (defecatory, urinary, and sexual) outcomes should be assessed in future research by using standardized and validated scoring systems.

Weak recommendation, low quality of evidence (GRADE 2 C).

Strength of consensus: 97%

*Statement 10.3:* Technological and training research:

- Technological innovations (e.g., haptic feedback, AI-guided systems) should be investigated in order to assess whether they are easy to apply and whether they bring significant technical advantages and clinical benefits in the use of robotic platforms for rectal prolapse surgery.
- Different robotic platforms could be compared for rectal prolapse surgery in order to identify whether they can be considered alternatives or if there are some clear indications for application.
- Studies assessing the surgeon’s learning curve and the efficacy of training programs integrating robotic rectal prolapse surgery should be conducted.

Weak recommendation, low quality of evidence (GRADE 2 C).

Strength of consensus: 100%

## Conclusion

This Delphi consensus provides a comprehensive and evidence-based framework for RVMR, addressing critical aspects of preoperative assessment, surgical indications, technical execution, and training. By standardizing practices and integrating multidisciplinary expertise, these recommendations aim to improve patient outcomes, reduce variability, and promote the safe and effective adoption of RVMR. Key findings highlight the importance of thorough preoperative workups, tailored surgical strategies, and structured training programs to minimize complications and optimize results. While robotic platforms offer significant technical advantages, cost-effectiveness, and resource allocation remain pivotal considerations.

While this is a pan-European consensus, local implementation should reflect national governance, multidisciplinary pathways, and guidance. In the UK, for example, practice has been shaped by the Cumberlege “First Do No Harm” report, which emphasizes robust preoperative multidisciplinary assessment and strengthened consent processes for procedures involving mesh. The principles in this document are consistent with those frameworks and intended to be applied with appropriate local adaptation.

Looking ahead, the panel emphasizes the need for robust, long-term studies to evaluate clinical efficacy, patient-centered outcomes, and technological innovations. Future research should also explore the potential of emerging robotic platforms and artificial intelligence in advancing precision surgery. Given that the Delphi method aggregates expert judgment in fields where high-quality data are limited, most of the recommendations rely on evidence of relatively low quality. Additional limitations include potential selection bias among panelists, the absence of patient representatives, and a lack of external validation. Future prospective studies and RCTs are needed to corroborate these consensus statements. This Delphi consensus strove to bridge evidence gaps, standardize clinical practices, and provide a roadmap for optimizing outcomes in RVMR.

## Supplementary Information

Below is the link to the electronic supplementary material.

ESM 1  (DOCX 15.3 KB)

## Data Availability

No datasets were generated or analysed during the current study.
