# Supplementary material for: 2025 Delphi consensus on robotic ventral mesh rectopexy
Source: Int J Colorectal Dis. 2025 Oct 16;40(1):214. doi: 10.1007/s00384-025-05003-8 (PMC12532664; doi:10.1007/s00384-025-05003-8)
Supplement: Supplementary file 1 — (DOCX 15.3 KB) [file 384_2025_5003_MOESM1_ESM.docx]

**Supplemental material**

**Statements Lacking Consensus**

During the Delphi process, two statements, 1.3 and 6.3, did not achieve consensus among the expert panel.

Statement 1.3 proposed the inclusion of neurophysiological testing, such as pelvic floor electromyography and somatosensory-evoked potentials, for patients with rectal prolapse and suspected neurological disorders.

*“Statement 1.3: Neurophysiological testing, including pelvic floor electromyography, sacral reflex latency, motor-evoked potentials, and somatosensory-evoked potentials, may be considered in patients with rectal prolapse and central or peripheral neurological diseases.*

*Weak recommendation, low quality of evidence (GRADE 2C)*

*Strength of consensus (1^st^ Delphi round): 68%*

*Agreement to delete statement 1.3 (2^nd^ Delphi round): 85%”*

The panel deemed the evidence insufficient, with no clear consensus on its clinical relevance or feasibility in routine practice. Consequently, this statement was deleted after the second round of discussion to streamline recommendations to clinically actionable and evidence-based guidelines.

Statement 6.3 addressed the lack of a universally optimal fixation technique for robotic ventral mesh rectopexy.

*“Statement 6.3: Any fixation technique can be considered for RVMR.*

*Weak recommendation, very low quality of evidence (2D)*

*Strength of consensus (1^st^ Delphi round): 67%*

*Strength of consensus (2^nd^ Delphi round): 60%”*

Although the statement aimed to provide flexibility in surgical approaches, panelists highlighted the variability in outcomes and the absence of robust comparative evidence supporting specific techniques. Despite revisiting the topic during the second Delphi round, agreement could not be reached. Ultimately, the decision was made to exclude this statement from the final recommendations, emphasizing the need for further research to establish best practices in fixation techniques.
